# Health economics education in undergraduate medical training: introducing the health economics education (HEe) website

**DOI:** 10.1186/1472-6920-13-126

**Published:** 2013-09-13

**Authors:** Raymond Oppong, Hema Mistry, Emma Frew

**Affiliations:** 1 Health Economics Unit, School of Health and Population Sciences, University of Birmingham, Edgbaston, Birmingham B15 2TT, UK; 2 Warwick Evidence, Warwick Medical School, University of Warwick, Coventry CV4 7AL, UK

**Keywords:** Medical students, Health economics, Online educational resource, Website

## Abstract

In the UK, the General Medical Council clearly stipulates that upon completion of training, medical students should be able to discuss the principles underlying the development of health and health service policy, including issues relating to health economics. In response, researchers from the UK and other countries have called for a need to incorporate health economics training into the undergraduate medical curricula. The Health Economics education website was developed to encourage and support teaching and learning in health economics for medical students. It was designed to function both as a forum for teachers of health economics to communicate and to share resources and also to provide instantaneous access to supporting literature and teaching materials on health economics. The website provides a range of free online material that can be used by both health economists and non-health economists to teach the basic principles of the discipline. The Health Economics education website is the only online education resource that exists for teaching health economics to medical undergraduate students and it provides teachers of health economics with a range of comprehensive basic and advanced teaching materials that are freely available. This article presents the website as a tool to encourage the incorporation of health economics training into the undergraduate medical curricula.

## Background

The UK health service is currently undergoing the most radical organisational change in history. Under the new structure, General Practitioners (GP’s) and other clinicians will have full responsibility for commissioning health care services, operating within Clinical Commissioning Groups (CCGs). It will be the GP’s responsibility to commission services to ensure that better outcomes are achieved whilst maintaining value for money. In the UK it has been strongly advocated that with this increased financial responsibility, doctors require training in understanding the economics inherent in health care to understand the implications of economic policy, guidelines and evidence based practice [1-5]. This need to incorporate economic training into undergraduate medical training is not unique to the UK. In other countries such as the US and Germany, it has been proposed that providing high-value, cost-conscious care is to become a core competency for training doctors [6,7]. Whilst there appears to be universal agreement that health economics should form an integral part of medical undergraduate training, there remains considerable diversity across medical schools in the UK in terms of the content of the health economics education and in the way the teaching is delivered [2].

As well as the consensus to have health economics training embedded into undergraduate medical curriculum, there is also an agreement that ideally this training should be provided by health economists [2]. However there is a shortage of health economics expertise within the UK capable of meeting all the training needs [8]. One compromise would be to make more use of online technology to aid teaching of health economics by both health economists and non-health economists. A recent study in the UK showed that health economics in medical schools was delivered by both health economists and public health professionals [2]. With the innovations in information technology there is now an increase in the use of web-based tools in education. In a recent review, of the learner use of online educational resources (OERs) for learning, Bacsich and colleagues recommended ‘in course redesign, institutions should aim to make more use of OERs and externally provided free-of charge, non-open resources in future programmes’ [9]. This paper presents our contribution in the form of the Health Economics education (HEe) website [10] as a toolkit to encourage the incorporation of health economics training into undergraduate medical training.

## Methods

### Design objectives

We set out to develop an online resource to provide teachers with materials to facilitate the teaching of health economics for medical students. We aimed to develop a website that: 1) encourages the teaching and learning of health economics; 2) supports teaching and learning in health economics; 3) creates a community of health economics teachers willing to share ideas, resources and expertise; and 4) promotes health economics to potential students.

To guide the development of the website we put together an advisory group made up of individuals actively teaching undergraduate and postgraduate health economics, members from the Economics Network and the Health Economists’ Study Group (HESG) – a large organisation of health economists in the UK as well as lay members. Together with the advisory board we identified the main ‘user-groups’ of the OER and it was clear from the outset that teachers of health economics to medical students would be one distinct user-group.

We aimed to put together a collection of online teaching resources that teachers of health economics could use regardless of their health economics training, so in theory, using this OER, non-health economists could teach health economics to medical students. To provide a resource that is easy to navigate, we decided to organise all the teaching resources by key topic areas within health economics. The topic areas were chosen based on the previous experience of teaching and directing health economics courses and generally accepted topic areas within the discipline. This meant that if a teacher of health economics wants to find resources to help with the development of a course on the basic principles of health economics they can access the website, click on the link for ‘introduction to health economics’ and be taken direct to all the OERs that is available for that topic area. The aim was to have available for each topic, a combination of up-to-date lecture slides, audio-visual material, group exercises, assessment material and reading lists which can be accessed freely.

### Step 1: collection of lecture slides, group exercises and assessment material

To obtain up-to-date teaching resources we made use of the close community of health economists working within the UK that are all members of the HESG. The HESG meet twice a year to discuss and debate methodological developments in health economics and through this network (and by personal communication) we were able to identify the course directors for all the health economics training courses that are offered across the UK. An email was sent to these course directors, explaining the rationale behind the website and inviting them to submit information about their courses in health economics and also to share the teaching material that they use. As well as this email request, we presented the development of the website at two HESG conferences with the aim of advertising and spreading the word about this initiative and encouraging teachers to share resources.

### Step 2: development of audio-visual resources

Recognising the need for material to help non-health economists teach health economics we submitted a research grant to the Economics Network to ask for funding to support the development of a series of audiovisual podcasts. The purpose of this part of the website was to have available downloadable audiovisual podcasts of experienced health economists talking about key topic areas within health economics. Non-health economics teachers can then download the material and insert them into lectures and use them as a series of ‘sound-bites’ to provide an understanding of the principles of health economics and to generate discussion within the classroom.

### Implementation

**Figure 1:**
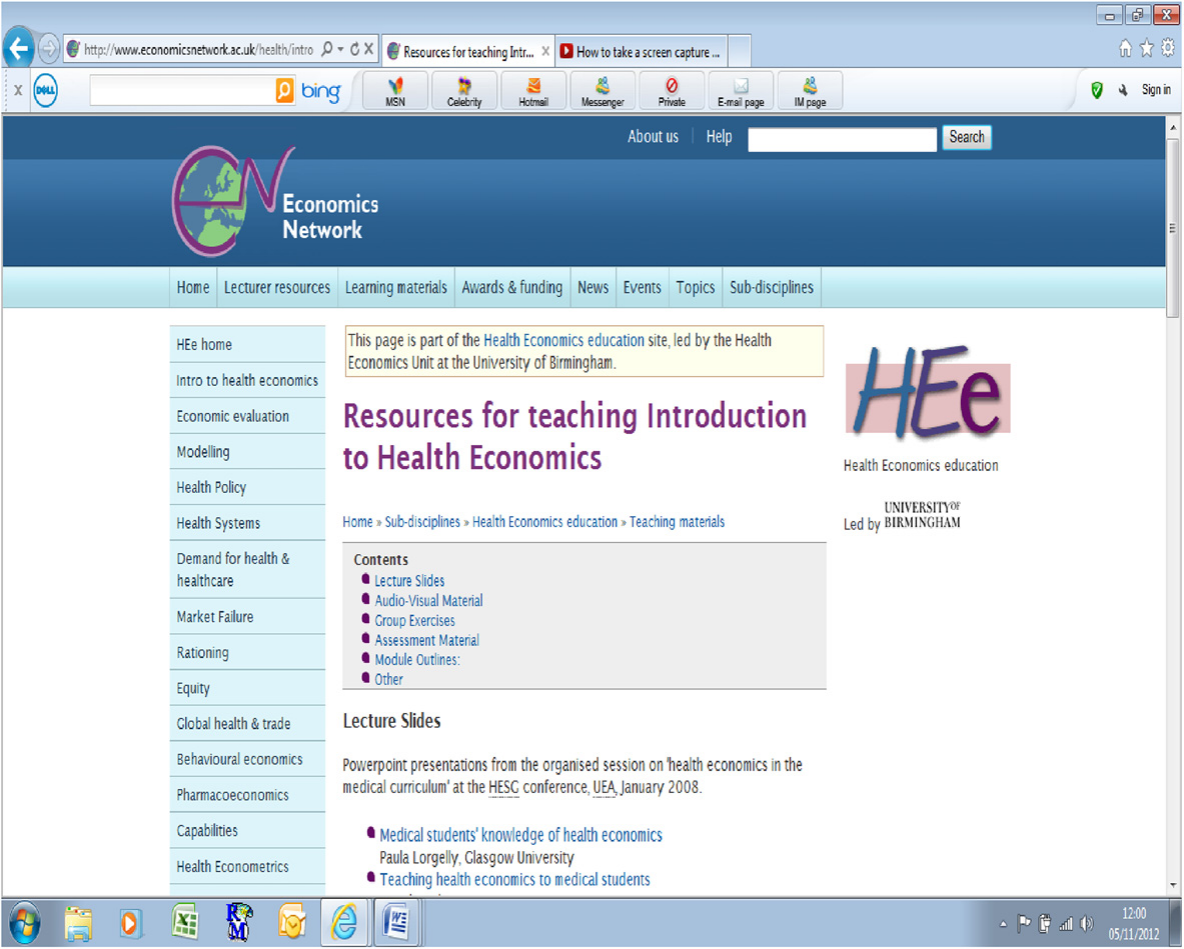
Screenshot of resources for teaching introduction to health economics.

The website now contains online teaching resources on a wide range of health economics topics. It is designed to enable users to navigate easily to teaching material relevant to the desired topic area and level of training. The following topics are covered: Introduction to health economics; Economic evaluation; Modelling; Health policy; Health systems; Demand for health and healthcare; Market failure; Rationing; Equity; Global health and trade; Behavioural economics; Pharmacoeconomics; Capabilities; and Health econometrics. When the user clicks on the desired topic area they are then taken to a complete listing of all available teaching resources. For example, within the topic ‘Introduction to health economics’, the following resources are freely available to download: lecture slides from 9 lectures focused on introducing the principles of health economics; 1 audiovisual podcast about the use of qualitative methods within health economics (with Professor Joanna Coast, University of Birmingham); an example of a game that can be used in the form of a group exercise to introduce students to the concepts of demand/supply, equilibrium and consumer/producer surplus; sample assessment questions from 5 exams; and examples of module outlines to provide the user with guidance on what sort of topics should be covered in an introduction to health economics course/module. The following image displays the user interface (Figure 1) for this section of the website. The complete list of topics always remains on the user bar on the left-hand side of the screen to enable the user to easily navigate between the topic areas around the website.

**Figure 2:**
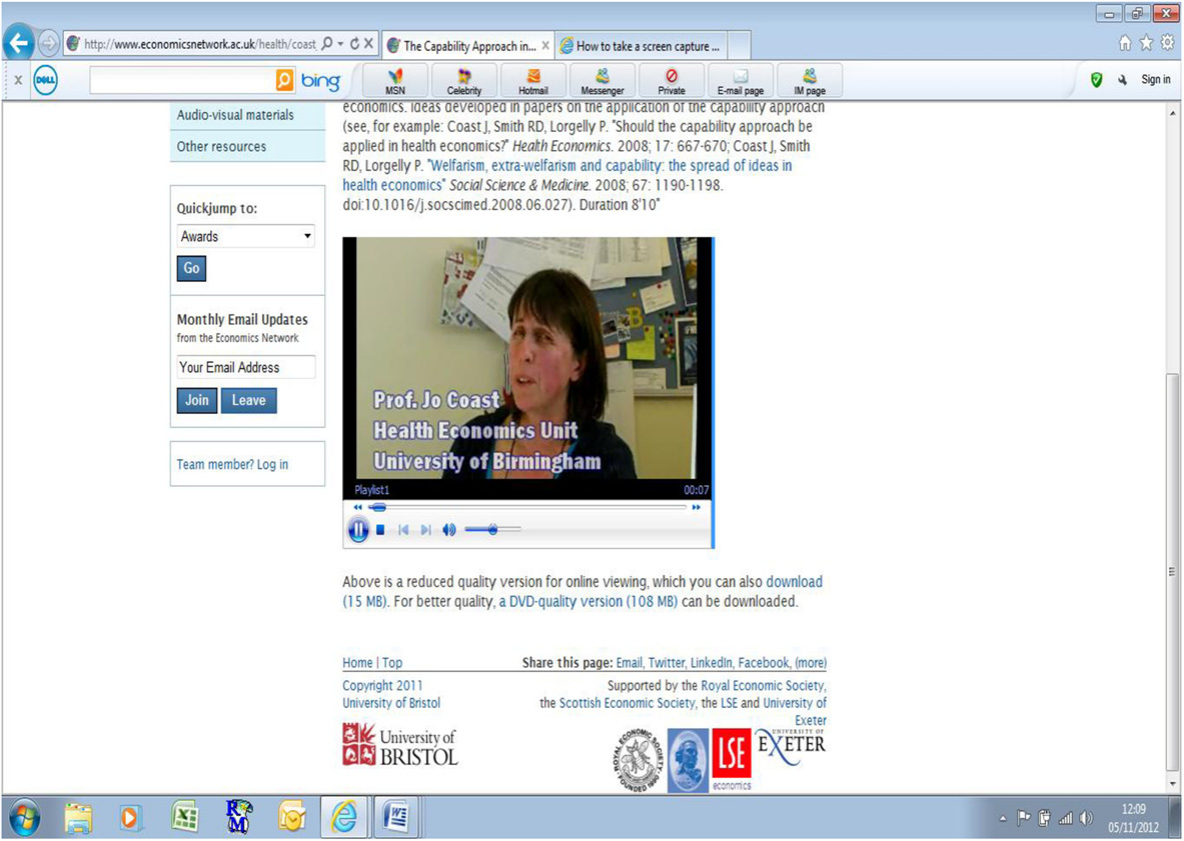
Screenshot of a sample audiovisual podcast.

For non-health economists teaching health economics to medical students, a potentially useful part of the website is the availability of the audiovisual podcasts. These podcasts can be used for two purposes. First, to provide the teacher with a useful background to the topic area before teaching it to medical students and/or second, to have available to download within lectures to provide medical students with the opportunity to listen to a health economist discuss their area of expertise. All of the podcasts have been developed using an ‘interview’ format with common questions answered by the expert. The running time of each podcast varies and ranges from 4 to 29 minutes. When the user clicks on the link to the podcast, they will be taken to a screen that provides a short description of the video with the option of either downloading the full size or a smaller size version. By clicking on one of these versions, they will then be taken to a screen where they can watch the video (Figure 2). There are 10 podcasts freely available to download on the website and Table 1 provides an outline of topic areas that are covered.

**Table 1:** Interviews available as downloadable podcasts

**Interviewee**	**Institution**	**Topic area**
Prof Cam Donaldson	Glasgow Caledonian University	Social business, health and wellbeing
Prof John Appleby	King’s Fund, London	GP commissioning
Dr Karen Bloor	University of York	Medical labour markets
Dr Tessa Peasgood	University of Sheffield	Wellbeing and health markets
Prof Mark Sculpher	University of York	Economic evaluation to support decisions
Prof Joanna Coast	University of Birmingham	Role of qualitative methods in health economics
		Limitations of QALYs
		Capabilities
		Possible disutility associated with explicit health care rationing
		The economics of antimicrobial resistance
Prof Alan Maynard	University of York	GP contracts
Prof Matt Stevenson	University of Sheffield	Modelling within health technology assessment

**Figure 3:**
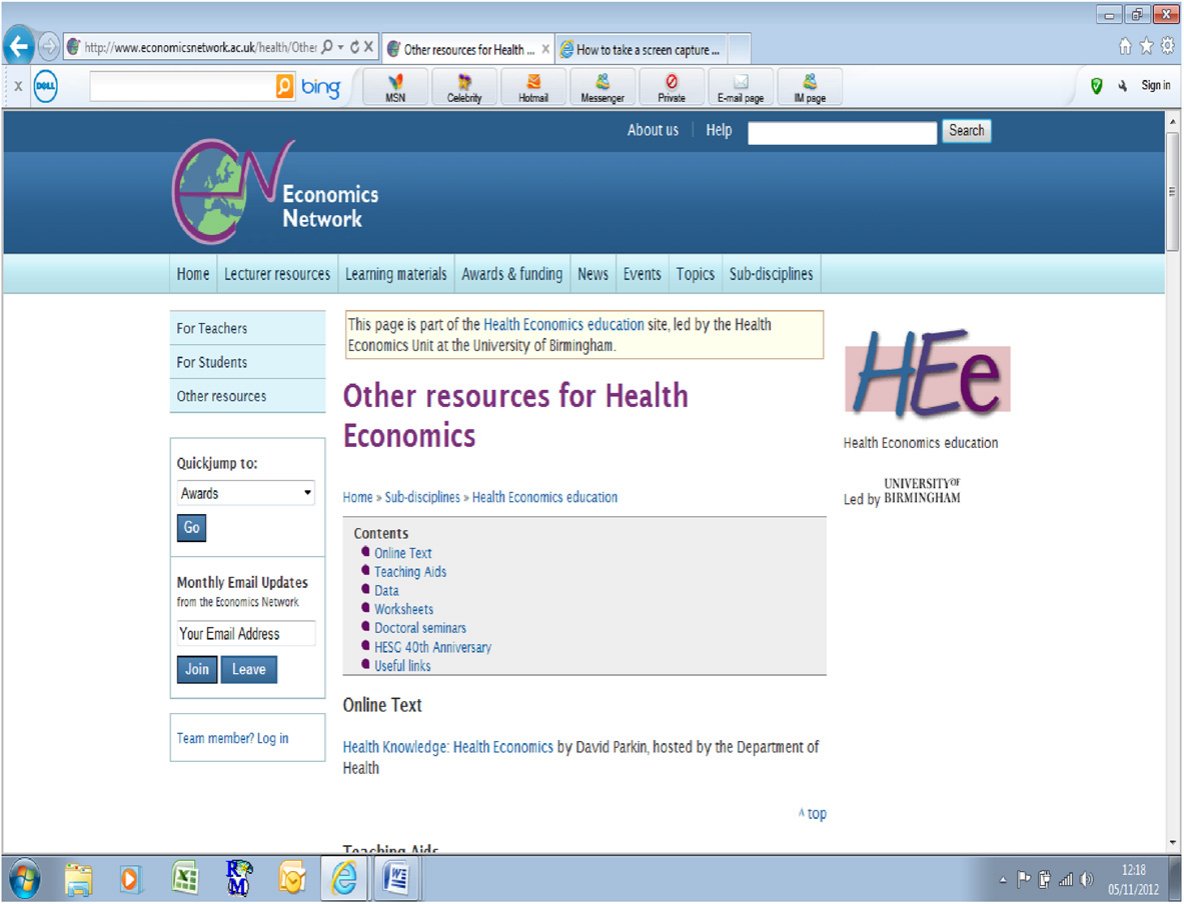
Screenshot of ‘Other Resources’ section of website.

Across the full website, users have access to 47 lectures, 11 examples of group exercises and 7 sample exam papers (for different levels) that are all freely available to download. In addition, there is suggested reading material also organised by topic and links to useful websites, up-to-date policy material and textbooks some of which have linked online educational support material. There is also an ‘Other Resources’ section on the website that contains additional information and tools to facilitate teaching. In this section there are useful datasets for students to practice with, EXCEL spreadsheets with examples of calculations e.g. how to bootstrap data to obtain confidence intervals, and information about seminars and conferences on related subjects. Figure 3 displays a screen shot of this section of the website.

## Discussion

Understanding the principles of health economics must be a core component of undergraduate medical training to prepare future doctors for managing health care expenditure. Current organisational reform within the UK health service has made this need for training even more important. This article presents an educational tool to facilitate the teaching of health economics to medical students driven by the desire to increase awareness of health economics amongst tomorrow’s doctors. The resource offers freely available downloadable teaching material to facilitate the teaching of health economics to medical students by non-health economists. Of course the website is also a valuable asset for health economists who are active in teaching; it provides a source to keep abreast of topical issues in health economics and provides a link to key policy documents. For the purposes of undergraduate medical training however, its primary value is to provide material to facilitate teaching by non-health economics experts.

Whilst the website is now an asset for teaching health economics, a number of challenges remain for it to achieve its full potential. Firstly, encouraging health economics lecturers to share teaching material and contribute to the website was a challenge. Plausible reasons for this could be the competitive nature of health economics training across the UK and the need for higher education institutions to retain a competitive edge when attracting students to study health economics. Also, as the website is newly developed, there is an uncertainty associated with it as teachers are only just becoming aware of it and learning about it’s objectives. We have made efforts to market its existence at conferences such as the Health Economists’ Study Group meetings (at Bangor (June 2011) and at Oxford (June 2012)) and at the European Conference on Health Economics (ECHE) meeting at Zurich in July 2012. It is our belief that with time as the website becomes more established through development and repeated advertising at key health economics events, teachers will become more familiar with it, engage in the initiative and become willing to share resources. Lastly, the use of online educational resources such as the HEe website have their shortcomings [11], and we acknowledge that formal training in health economics might still be required by non-health economists who are engaged in teaching medical students.

We believe that the website is now an excellent resource for new teachers to health economics as it contains a diversity of online resources. These resources can be used to deliver teaching in a didactic fashion or to enhance learning through the use of group exercises and audiovisual material. It now exists as a potentially valuable resource to provide health economics training to medical students.

## Abbreviations

HEe: Health economics education; OER: Online education resource; GP: General practitioner; CCG: Clinical commissioning group; HESG: Health economists study group.

## Competing interests

The authors declare that they have no competing interests.

## Authors’ contributions

All authors contributed extensively to this piece of work. RO, HM and EF contributed to redesigning the website. EF supervised the project and provided advice. RO and HM gathered information for the website and carried out the updating of the website. RO produced the first draft of the paper. All authors contributed to drafting the paper and provided comments on the paper throughout the whole process. All authors approved the final version of the paper.

## Pre-publication history

The pre-publication history for this paper can be accessed here:

http://www.biomedcentral.com/1472-6920/13/126/prepub
